# Further understanding human disease genes by comparing with housekeeping genes and other genes

**DOI:** 10.1186/1471-2164-7-31

**Published:** 2006-02-21

**Authors:** Zhidong Tu, Li Wang, Min Xu, Xianghong Zhou, Ting Chen, Fengzhu Sun

**Affiliations:** 1 Molecular and Computational Biology Program, University of Southern California, Los Angeles, California 90089, USA

## Abstract

**Background:**

Several studies have compared various features of heritable disease genes with other so called non-disease genes, but they have yielded some conflicting results. A potential problem in those studies is that the non-disease genes contained a large number of essential genes – genes which are indispensable for humans to survive and reproduce. Since a functional disruption of an essential gene has fatal consequences, it's more reasonable to regard essential genes as extremely severe "disease" genes. Here we perform a comparative study on the features of human essential, disease, and other genes.

**Results:**

In the absence of a set of well defined human essential genes, we consider a set of 1,789 ubiquitously expressed human genes (UEHGs), also known as housekeeping genes, as an approximation. We demonstrate that UEHGs are very likely to contain a large proportion of essential genes. We show that the UEHGs, disease genes and other genes are different in their evolutionary conservation rates, DNA coding lengths, gene functions, etc. Our findings systematically confirm that disease genes have an intermediate essentiality which is less than housekeeping genes but greater than other human genes.

**Conclusion:**

The human genome may contain thousands of essential genes having features which differ significantly from disease and other genes. We propose to classify them as a unique group for comparisons of disease genes with non-disease genes. This new way of classification and comparison enables us to have a clearer understanding of disease genes.

## Background

Identification of novel genes associated with human diseases is among the most critical tasks in medical research. Towards this goal, various features have been compared between heritable disease genes and non-disease genes [1-4]. Although most findings were consistent with each other, a few conflicting results showed up. For example, Smith et al. [3] found that disease genes evolved with higher nonsynonymous/synonymous substitution rate ratios (Ka/Ks) than non-disease genes, but Huang et al. [4] found no such significant differences. One common problem with these studies is that human essential genes were ignored and simply grouped together with other non-disease genes. Essential genes are genes whose functions are necessary for the organism to survive and reproduce. Since the disruption of essential genes' function will cause fatal consequences, they should be regarded as the most severe "disease" genes. Therefore, comparing disease genes to a mixture of essential and non-disease genes will reduce the clarity of the signals of the disease-related features and may even lead to erroneous findings. Thus, it is beneficial to separate human essential genes from other non-disease genes before comparisons are made.

Thousands of genes have been identified as essential genes in multiple model organisms, such as *Saccharomyces cerevisiae*, *Caenorhabditis elegans*, and *Mus musculus *[5-7]. Although it is almost certain that the human genome also contains hundreds to thousands of essential genes, it's impractical to experimentally determine them as in *S. cerevisiae *or *C. elegans*. The absence of a set of well-defined human essential genes poses a challenge on studying them and urges for alternative solutions.

The human genome has an extremely complex tissue expression profile. Some genes are expressed only in certain tissues during specific times, while others are constitutively and ubiquitously expressed [8,9]. For the latter genes, they are presumed to be necessary for the most fundamental cellular physiological processes and are referred as housekeeping genes [9]. Housekeeping genes have been studied by many researchers and some interesting observations have been reported. For example, Zhang and Li found that housekeeping genes evolved more slowly than tissue-specific genes [10]. Eisenberg and Levanon found that housekeeping genes were compact in their coding lengths, which could be the result of higher selective pressure[11]. Based on the unique properties of the ubiquitously expressed human genes (UEHGs), we believe that they are suitable candidates for essential genes. Although this hypothesis is intuitive and sounds reasonable, serious efforts are required to collect supportive evidence on a systematic level.

In this study, we consider a set of 1,789 ubiquitously expressed human genes (UEHGs) as an approximation for essential genes. We demonstrate that UEHGs are very likely to contain a large proportion of essential genes and thus can approximate human essential genes. By performing a three-way feature comparison of UEHGs (presumed essential genes), disease genes, and the rest of human genes (referred as other genes), we show that they are different in many aspects such as the evolutionary conservation rates, DNA coding lengths, gene functions, etc.

## Results

Instead of dividing the human genome into disease vs. non-disease genes, we choose a three-way classification, namely, UEHGs (presumed "essential"), disease, and other genes. We first validate that the set of UEHGs contains a large fraction of essential genes. Then by comparing the three groups of genes, we see how the disease genes can be distinguished from essential and other genes. If UEHGs really contain much greater fraction of essential genes than non-UEHGs (i.e. disease and other genes), we expect to observe the followings. First, as essential genes are functionally extremely important, the selective pressure on them are much higher than on non-essential genes, thus UEHGs should have a slower evolutionary rate than both disease and other genes [12,13]. Second, since most Mendelian diseases are caused by deleterious amino acid substitutions, if we study the conservation at amino acid level, we expect to see different patterns for UEHGs, disease and other genes. Third, when UEHGs are mapped to another species, the homologous genes should more likely be essential in that species if the species is evolutionarily close to humans. Fourth, since essential proteins usually tend to be hub proteins (highly connected) in the protein-protein interaction network [14], UEHGs should have a higher average physical interaction degree than non-UEHGs. Fifth, the functions of UEHGs should be fundamentally important. To verify these hypotheses, we compile the lists of UEHGs, disease genes and other human genes. We then collect various features and compare those selected features among the three gene classifications.

### Comparison on the evolutionary features

**Figure 1:**
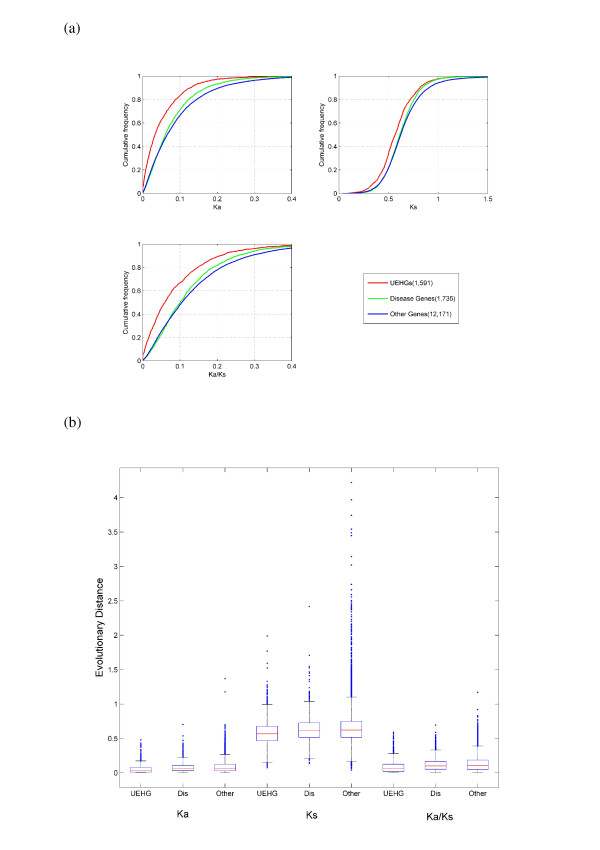
**Distribution of Ka, Ks and Ka/Ks **(a) The cumulative density of Ka, Ks and Ka/Ks derived from human-mouse orthologous pairs. Ka, the number of non-synonymous substitutions per non-synonymous sites. Ks, the number of synonymous substitutions per synonymous site, and the Ka/Ks ratio. Three groups of human genes are represented in different colors and the number of genes in each group is listed right to the line symbols. (b) The box plots are drawn based on the same data. For each category, the central box depicts the middle 50% of the data between the 25th and 75th percentile, and the enclosed red horizontal line represents the median value of the distribution. Extreme values are indicated by solid blue dots that occur outside the main bodies of data.

We first compare the Ka, Ks and the ratio (Ka/Ks) based on the three-way classification of the human genome. The Ka, Ks and Ka/Ks are derived from both human-rat and human-mouse orthologous pairs. The results obtained from human-mouse orthologous pairs indicate that UEHGs have the smallest Ka, Ks and Ka/Ks ratio in the three groups (P-values for UEHGs vs. disease are 3.4E-39, 6.3E-15, and 1.7E-38; P-values for UEHGs vs. others are 5.3E-64, 9.0E-27, and 1.3E-57, respectively for Ka, Ks and Ka/Ks), and disease genes have lower evolutionary rates than other genes (P-values are 9.1E-5, 5.5E-4 and 2.6E-4 for Ka, Ks and Ka/Ks, respectively) (Fig 1). By various statistical measurements, UEHGs consistently stand out as the slowest evolved gene group and the difference between UEHGs and the other two groups is greater than the difference between disease genes and other genes (Table 1). The results are similar when human-rat orthologous pairs are used to calculate Ka and Ks, only the P-values are slightly less significant. Again, disease genes evolve at slower rates than other genes with significant differences in Ka, Ks and Ka/Ks (P-values are 0.008, 0.052 and 0.026, respectively). UEHGs evolve at the slowest rates and the differences in Ka, Ks and Ka/Ks are strongly significant (P-values for UEHGs vs. disease are 7.0E-32, 3.2E-13, and 2.7E-28; P-values for UEHGs vs. others are 1.1E-49, 4.8E-23, and 1.2E-44, respectively, for Ka, Ks and Ka/Ks) (Fig S2). Here, our results are different from the findings of Smith [3] and Huang [4], and as described before, their results are not consistent with each other either. We think this is partly due to the effect of mixing essential genes with non-disease genes in the previous studies. Another reason is that different groups used different set of disease and non-disease genes (Smith et al. studied 387 disease and 2,024 non disease genes, Huang et al. studied 1,112 disease genes and more than 10,000 non-disease genes and our dataset covered >1,700 disease genes and >12,000 non-disease genes, see supplementary materials for a detailed comparison). Our results indicate that UEHGs are under the strongest selection pressure. Disease genes evolve at an intermediate rate which is slower than other genes, but faster than UEHGs. Our experiments consider much larger gene sets and a significant number of essential genes are separated out from non-disease genes. Therefore, our results may better reflect the relationship of disease genes with other genes. The slightly different results calculated from rat and mouse are most likely due to the different evolutionary rates between mouse and rat after their divergence around 16 million years ago [15]. We also confirm the positive correlation between Ka and Ks in all the three gene groups, as observed in previous studies [16-20]. For example, the Ka and Ks of disease genes have a correlation coefficient of 0.45 (P-value is 1.9E-86 by t-test). Although a synonymous mutation has no apparent effect on protein sequence, it may affect the DNA structure, mRNA structure, and biological processes such as transcription and RNA splicing [21]. The small average Ks of UEHGs indicates that the synonymous sites of UEHGs are also under stronger selection than the other two groups, which further suggests the functional importance of UEHGs.

**Figure 2:**
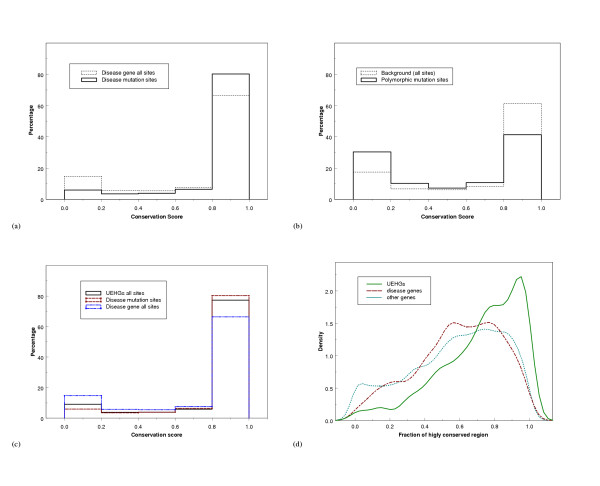
**Codon conservation of the three gene groups. **The conservation score of amino acids of the three groups of genes are compared. (a) The distribution of disease causing mutation sites' conservation score is plotted in the solid line. The dotted line is drawn based on the conservation scores of all the sites in the coding region (i.e., the distribution of the conservation score when sites are randomly chosen). (b) The distribution of polymorphism mutation sites' conservation score vs. the random distribution as in (a). (c) The distribution of conservation score for UEHGs (black line), disease gene (broken blue line) and disease causing mutation sites (red broken line). (d) The distribution of the fraction of the highly conserved regions (Cons. Score>0.9). Each human gene group is represented in a different color.

As Ka and Ks are summary statistics of the nucleotide substitution rate of a gene, and most Mendelian diseases are caused by amino acid substitutions in coding regions, it will be more informative to study the pattern of conservation for individual nucleotides or amino acids. We obtain the conservation score of specific amino acids based on a large-scale multiple-sequence alignment of 8 species performed recently by the UCSC research group [22]. The conservation scores were derived from a two-state phylo-Hidden Markov Model and can be interpreted as probabilities of each base being from a conserved hidden state. We collect a list of more than 6,000 disease mutation sites and about 1,900 polymorphism (neutral) mutation sites from SwissProt. We compare the conservation of these two types of sites with each other and also with the background (The background is obtained by considering the conservation score of all the amino acids in coding regions). As shown in Fig 2 (a, b), polymorphism mutation sites are significantly biased towards less conserved sites while disease mutation sites are significantly biased towards more conserved sites (p-values < 10^-5 ^in both cases). This is consistent with the findings of Miller and Kumar although they focused only on seven disease genes [23]. Then we compare the UEHGs with disease genes and results are shown in Fig 2(c). UEHGs are more conserved than disease genes but the conservation score of disease mutation sites are greater than those for UEHGs. Since we don't have information on "essential sites", we are unable to directly compare the "essential sites" with disease sites. Instead, we think that one possible mechanism distinguishing essential genes from disease genes is that essential genes contain a larger fraction of highly conserved sites (with the underlying assumption that highly conserved sites correspond to functionally important loci). Thus, the chance that a random mutation will cause a severe phenotype will be much higher for essential genes than for disease genes. We select conservation score 0.9 as the cut-off value and define sites with conservation scores above that as highly conserved sites. The cut-off is chosen based on the distribution of the conservation scores of disease mutation sites. Different cut-off values were tested and results are similar. We calculate the fraction of highly conserved sites in the coding region and show the distribution of this fraction for UEHGs, disease genes and other genes in Figure 2(d). It's clear that UEHGs contains a much higher fraction of highly conserved sites than the other two groups, while there is no significant difference between disease genes and other genes (p-value = 0.32).

### Cross-species comparison of gene deletion phenotypes

**Figure 3:**
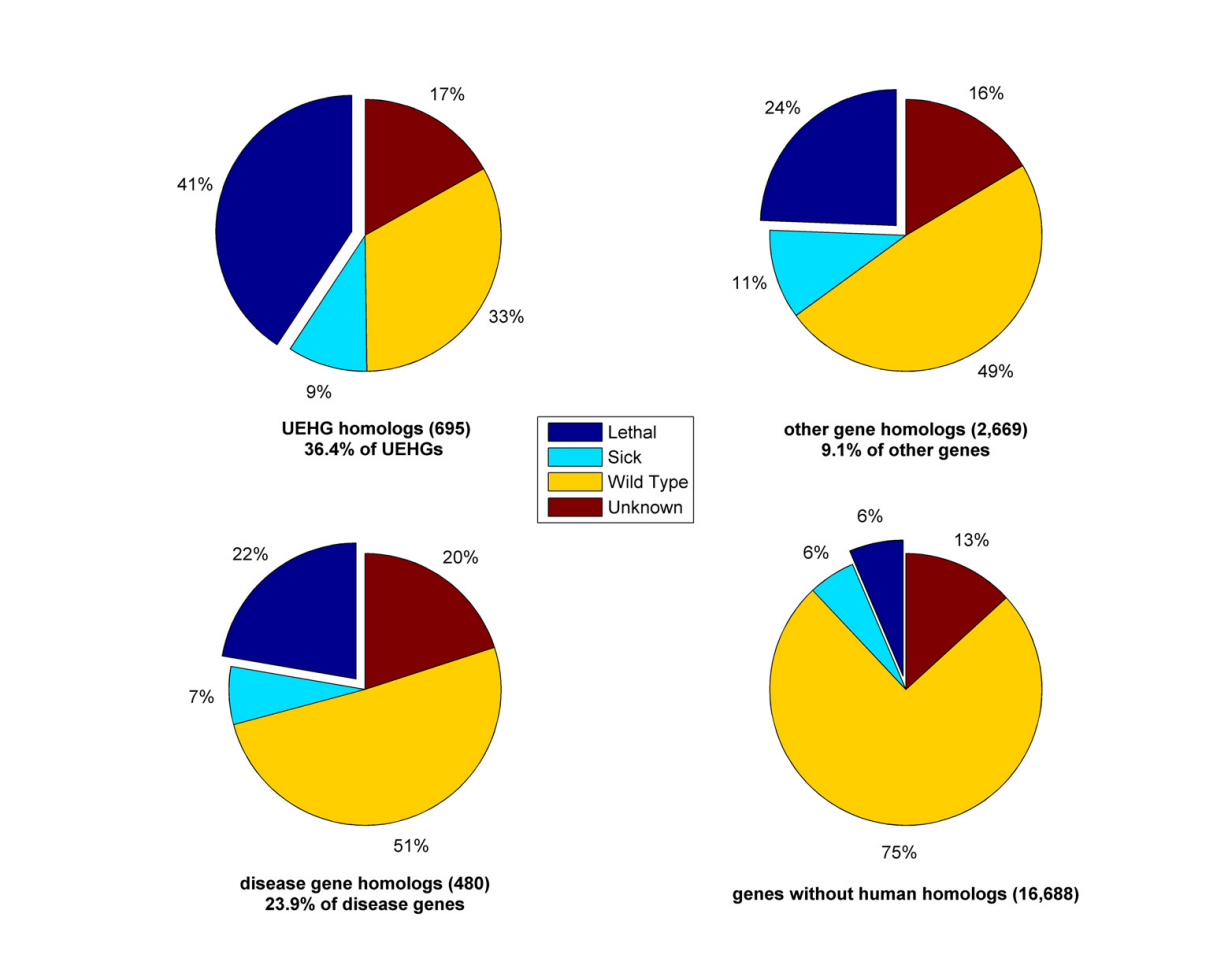
**Comparison of gene essentiality between human and *C. elegans ***Human genome is divided to three groups as described in the main text and 20,488 *C. elegans *genes are mapped to each group based on homology. The essentiality of *C. elegans *gene is obtained from RNAi-interference experiment as described in the main text. Different phenotypes are represented by different colors and the number of the homologs in each group is listed. The fraction of human genes with *C. elegans *homologs is shown under the group name.

Many human diseases are studied by experimenting on model organisms such as mouse. The underneath rationale is that the homologous genes have similar functions if the two species are evolutionarily close. Similarly, the essentiality of human genes can be tested in an evolutionarily close species. This approach may not work for all genes, due to differences between species, however, the closer the two species are, the higher accuracy it can achieve. Unfortunately, the knowledge on gene essentiality in other high animals is still very limited. For example, the number of mouse genes with known mutation phenotypes is around 10% of the genome (~2,800 in Mouse Genome Informatics database) and they are heavily biased towards the homologs of human disease genes (results not shown). By now, only *S. cerevisiae *and *C. elegans *have been explored for gene essentialities on the whole genome scale [5,6]. Here we compare the human genome with them, although they are not favoured for the rather far evolutionarily distances between them and humans. Human genes are mapped onto yeast and worm based on homologous relationship. For UEHGs, disease genes and other genes, we examine the fraction of genes which are homologous and essential in the mapped species (Table 2 and Fig 3). The first column of Table 2 shows that UEHGs contain the largest fraction of genes which have homologous counterparts in yeast. Disease genes have smaller fraction than UEHGs but greater than other genes. The same order can be observed in *C. elegans *as shown in Fig 3. Since yeast and *C. elegans *are evolutionary distant from human, the results support that UEHGs contain a greater fraction of functionally important genes than disease and other genes. The second column of Table 2 shows the fraction of homologous genes in each three groups which are also essential in yeast. Again, we see that UEHGs have the highest fraction, followed by disease genes, and the other genes. However, as shown in the same column, conditional on homologous genes, the fraction of essential genes for each group does not strictly follow the same order (i.e., UEHGs>disease genes>other genes). This is more prominent in yeast than in *C. elegans*. Therefore the phenomenon is very likely caused by the larger evolutionary distance between yeast and human. Or we can say that given a gene is highly conserved (such as a human-yeast homologous gene), the essentiality of the gene is no longer strongly linked to the group that the gene belongs to. In addition, the results suggest that the highly conserved genes contain large fraction of essential genes too.

### Comparison on other features

**Figure 4:**
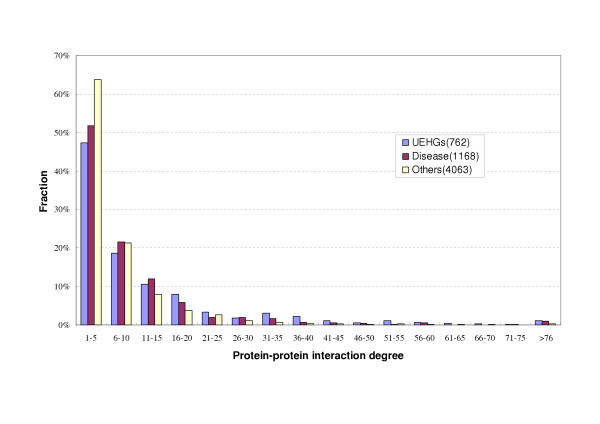
**Distribution of protein physical interaction degrees. **UEHGs, disease genes, and other genes are shown in three different colors in the histogram. It can be seen that as the interaction degree increases, the fraction of UEHGs also increases. For the summary statistics, see main text. The number of genes with at least one interaction in HPRD is listed for each gene group.

It has been noticed for several years that, in the protein interaction network, essential proteins tend to interact with more proteins in model organisms [14]. Peri et al. manually collected more than 27,000 interactions involving about 18,000 human proteins [24]. Although this data set is still quite sparse, its accuracy is assumed to be high. The distribution of protein physical interaction degree of the UEHGs, disease and all other genes are shown in Figure 4. The average degree ± standard error for UEHGs, disease and all other genes are 11.0 ± 0.6,8.4 ± 0.4 and 6.2 ± 0.1, respectively. We only consider proteins with at least one interaction, since 0 degree could mean either no interaction or absence of data and we are unable to make the distinction. We acknowledge that the data collection could be biased towards human disease genes. However, as the set of UEHGs are defined purely based on gene expression from an independent source, it's unlikely to have a heavy bias towards UEHGs. Thus, the high interaction degrees of UEHGs can be regarded as a supporting evidence for their essentiality.

**Figure 5:**
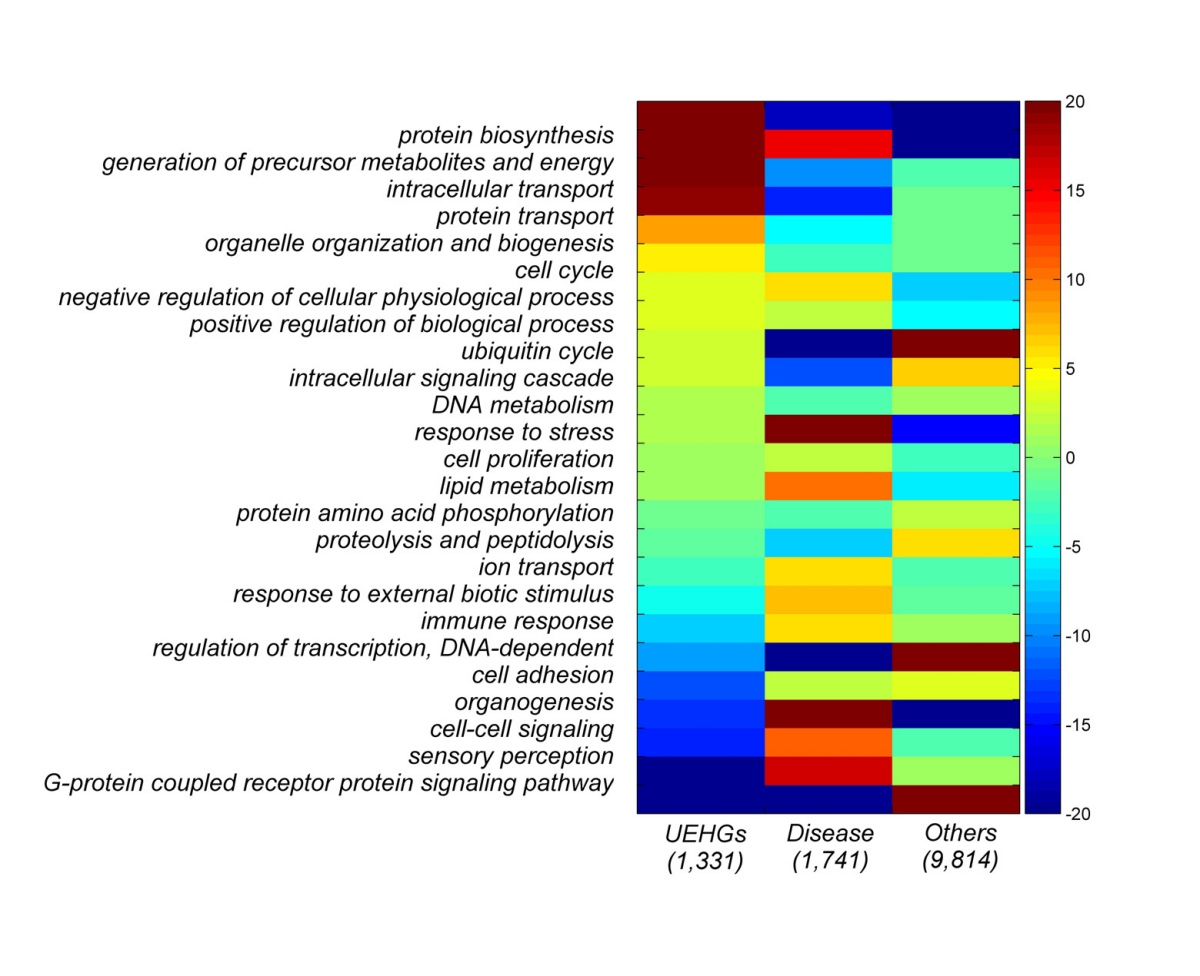
**Function annotation of genes in the three groups. **GO categories are described by the row labels and columns are the three classes of genes. A color scheme (scale shown on the right) is used to display the significance level of over-representation (numbered as negative logarithm of the P-value, upper half of the scale) or under-representation (numbered as logarithm of the P-value, lower half of the scale) for certain gene group and function category. Hyper-geometric distribution is used for the calculation of the P-value.

We also investigate the function annotation of the three groups of genes. As shown in Figure 5, UEHGs, disease genes and all other genes have distinct function distributions. UEHGs are enriched in protein biosynthesis and several other fundamentally important physiological processes, while disease genes are more relevant to sensing and responding to internal/external signals, which are advanced mechanisms for the fine tuning of certain biological processes.

**Figure 6:**
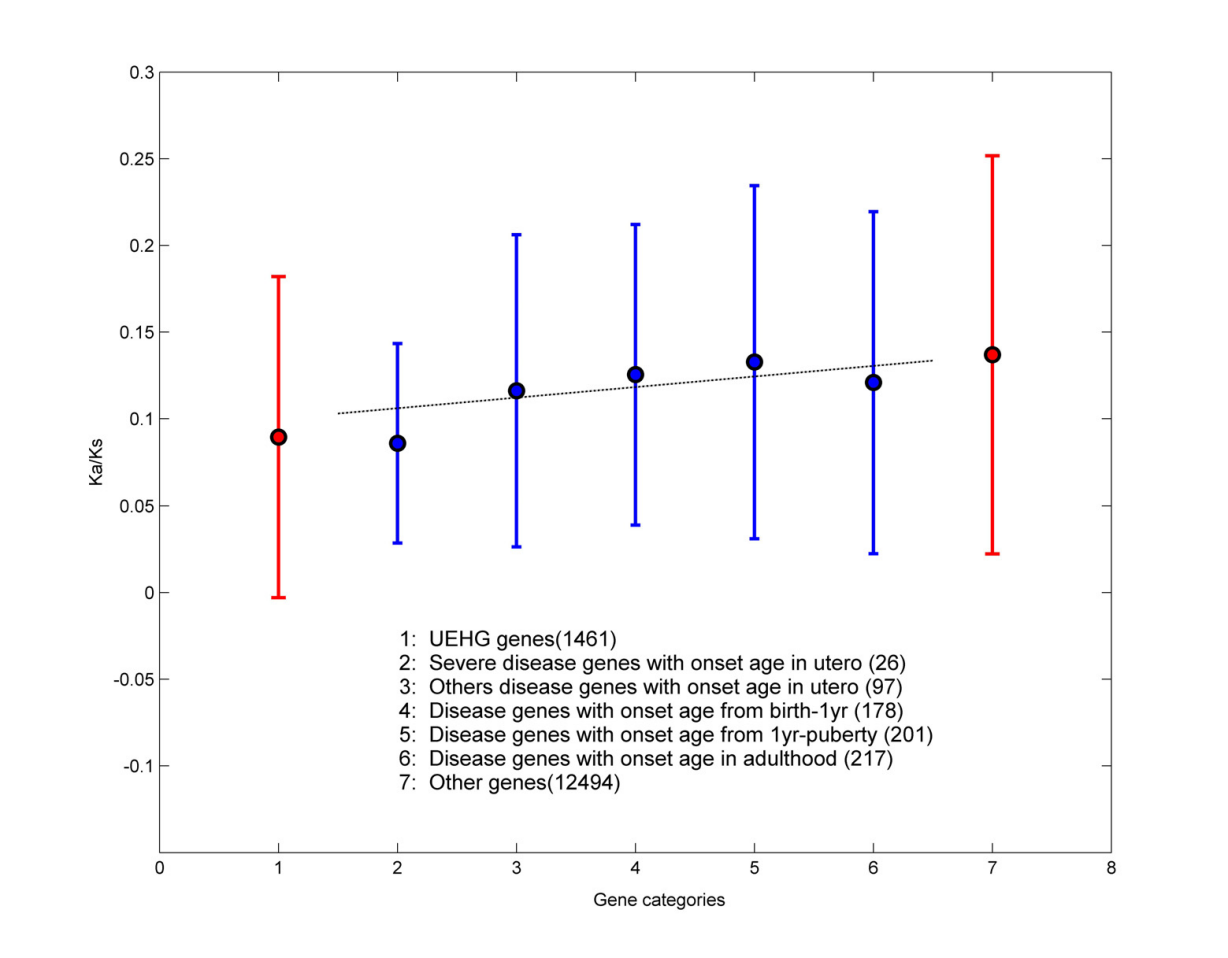
**Correlation of disease onset age with Ka/Ks. **The correlation of disease onset age with Ka/Ks. Disease genes are divided into 5 groups based on disease onset age. The weighted linear regression is applied to disease genes (group 2 to 5) and is shown as the dotted line. The coefficient for onset age is +0.0086 and P-value is 0.02, derived from the regression. UEHGs and other genes are plotted on the two sides of the diseases genes for visual comparison. The standard deviation is indicated by the short horizontal bar and mean is denoted by the solid circle. The large variation in each group hints for other confounding factors which also affect Ka/Ks.

Finally, we look at the relationship between gene's conservation and the onset age of disease. Different diseases exhibit their symptoms at different ages. Some diseases develop as early as in utero while some only present in elders. People usually think genes associated with early onset diseases are under higher selection pressure than those associated with late onset diseases. If this is correct, since essential genes are critical, their evolutionary rates should be similar to those early onset disease genes rather than the late onset ones. To verify this, we divide disease genes based on their onset age as Jimenez et al. [25] and compare the Ka/Ks ratio. Figure 6 shows that evolutionary rates tend to increase when the onset age becomes larger. The correlation coefficient between the onset age and the Ka/Ks ratio is positive with P-value of 0.02 based on weighted least squares regression [26]. (Weighted least square is used here since different age groups contain unequal number of genes with non-constant variances, by introducing weight to the regression, such effects can be reduced.) Base on visual inspection, Fig. 6 also suggests that UEHGs have similar Ka/Ks ratios as those for genes responsible for diseases in uterus and other genes have similar Ka/Ks ratios with genes associated with late onset diseases. However, as the regression is performed on the group number rather than the actual disease onset age (the original linear relationship among different disease onset ages could be distorted to some extent), and the P-value just passes a less stringent cut-off (i.e., 0.05), more data and further analysis are needed to draw a more confident conclusion from above results.

## Discussion

All the results above support that UEHGs by themselves form a distinct group other than disease genes. The results also endorse that UEHGs may contain a large proportion of functionally essential genes. Although we try to show that UEHGs are good candidates for human essential genes, we have no intention to claim that they are the only or the best gene set for representing human essential genes. Because a gene needs to be ubiquitously expressed to be considered an UEHG, low expressed or somehow tissue specific expressed essential genes will be excluded. Also, since the tissue samples were collected mainly from adult individuals, genes which are essential for early stage development may be missed too. As revealed by the cross-species comparison, UEHGs may have failed to cover many essential genes and those genes are still classified as other genes. We study a different set of genes by considering genes that are conserved across yeast, *C. elegans *and human. The results indicate that they may contain a large fraction of essential genes too (results not shown). However, as pointed out by Chervitz et al. [27], such set may miss many human essential genes which don't have homologs in yeast and *C. elegans*. In contrast, UEHGs is a more unbiased sample from all essential genes. A combination of UEHGs and conserved genes might generate a more complete set of candidates for human essential genes. We also realize that the set of disease genes in our study are mainly genes associated with Mendelian diseases, while complex disease genes are under-represented.

Different from previous studies on human housekeeping gene, we define the UEHGs as genes expressed in "almost all" (not "exactly all") the tissues that are examined. Due to the fluctuation of gene expression and the error in the gene expression measurement, as more tissues being examined, fewer genes will be observed as expressed in all the tissues. We relax the criteria to allow missing expression in a small fraction of tissues so that the size of UEHGs is less sensitive to the number of tissues being examined. Also a different cutoff value of expression level was adopted. In order to verify that our results are not sensitive to specific criteria used to define UEHGs, we prepare another UEHGs set defined as genes expressed at more than 300 standard units in all the 79 tissues. This leads to 2,038 genes being grouped as UEHGs, and 1,509 genes are contained in the original set of 1,789 genes. The evolutionary rates are compared among the new set of UEHGs, disease genes and other genes. The results are almost identical as before except for the slight changes in P-values (see details in supplementary materials). This indicates that our findings are not sensitive to the criteria for defining the UEHGs.

Previous studies have shown that house-keeping genes have shorter coding length [11] while disease genes usually have longer coding length [1]. We confirm these findings in a three-way comparison (Table 3). Since UEHGs are required to be expressed in all the tissues constitutively, it's beneficial to have the intron and untranslated regions shorter than other genes. But it is unclear why disease genes are generally longer than other genes. One possible explanation is that the functions of many disease genes can only be performed by proteins with certain lengths. For example, some ion channel proteins (e.g. cystic fibrosis transmembrane conductance regulator, CFTR) need to span through the membrane multiple times to form the pore structure, a task which can not be fulfilled by a short protein. Further studies are needed to explore how general such cases are.

We also want to point out that, as shown in Fig 6, there are no sharp dividing lines among essential genes, disease genes and other genes. Some diseases are simply lethal and the associated genes are essential genes by the definition. Some diseases have much less severe effects and it's hard to distinguish them from true non-disease genes. Thus, the gene essentiality might be better described by a continuous spectrum rather than by artificially divided groups. Even more complicated situations arise when different mutation forms are considered. Since different mutations usually lead to phenotypes of different severities [28,29], a disease gene could be either a non-essential gene or essential gene but with non-lethal mutation form. Thus, any simple grouping of human genome may lack the power for accurately illustration of the complex scenario associated with human disease genes.

## Conclusion

Our studies suggest that human essential genes are a unique group of genes and should not simply be ignored and classified with non-disease genes for the studies on disease genes. We also show that disease genes have several properties residing between essential and other genes. We notice that gene essentiality might better be described in a continuous spectrum instead of being assigned a class label. Nevertheless, the simplicity of the three-way classification is good for the purpose of this research since comparisons can be performed easily.

Extensive knowledge on human essential genes can be critical for the understanding of human diseases. It has been shown that essential genes may have direct association with diseases such as cancer [30,31]. Studying human essential genes might also provide key clues for questions such as how human beings evolved. However, limited attentions have been paid to them and very little systematic studies have been done. We showed how the picture of disease genes gets clearer when we explicitly consider the essential genes. We believe the updated global picture of disease genes will enable us to better identify them in the future [32].

## Methods

### Compiling lists of disease genes and UEHGs list

The list of disease genes were obtained from OMIM [29]. 3,962 records were listed in the morbidmap (Jun 6, 2005) and entries with known sequence (OMIM ID marked with *), with known sequence and phenotype (OMIM ID marked with #), and with phenotype description, molecular basis known (OMIM ID marked with +) were retained for this study. A total of 2,012 genes with unique OMIM Ids were finally collected as human disease genes.

Ubiquitously expressed genes were obtained from the result of a recent large scale microarray experiment on human gene expression patterns by Su et al. [33]. A total of 33,698 genes sampled from 79 tissues were interrogated in their experiments. The overall gene expression level was 776.5 standard Affymetrix average difference units, and genes with expression level greater than 550 standard units in at least 73/79 tissues were selected as UEHGs (a conservative estimation on the percentage of essential genes in the human genome is about 10%, thus the standards were set so that roughly 2,000 genes would be classified as UEHGs). A total of 1,789 such genes were collected. The set of UEHGs has a small overlap with disease genes as 176 genes belong to both classes. The full list of UEHGs can be found in Additional file 2.

### Collection of gene features

The mouse and rat homologs and corresponding synonymous substitution rate (Ks), nonsynomymous substitution rate (Ka) of totally 15,726 human genes were downloaded from NCBI HomoloGene [34]. To prevent possible contamination by paralogous genes, we only considered one-to-one mapped orthologous pairs. To test the statistical significance of the difference of Ka, Ks and Ka/Ks distributions among the three groups, Kolmogorov-Smirnov test was used to calculate the p-value as in [4] so that direct comparisons could be possible. Nucleotide conservation scores were downloaded from UCSC Genome Browser website [35]. Human sequence variation information was obtained from Swiss-Prot protein knowledgebase [36]. The original amino acid positions were mapped to nucleotide positions on the corresponding chromosome to obtain the conservation score. To study the correlation of the onset age of a disease with its conservation, we obtained the onset ages of over 900 genes from [25]. Weighted least square regression is used to find the correlations between disease onset ages and Ka/Ks ratios [26].

Yeast genes were collected from NCBI Entrez Gene Database [37] and were divided into four groups: UEHG homologs, disease gene homologs, other human gene homologs, and genes without human homologs. The homologies were obtained from NCBI HomoloGene as described above. The yeast gene deletion phenotype data were downloaded from *Saccharomyces *Genome Database [38]. Similarly, genes in *C. elegans *were collected from NCBI Entrez Gene Database and were divided into four groups. RNAi phenotypes of *C. elegans *genes were retrieved from WormBase [39]. The RNAi phenotypes were divided into four categories: lethal (including both embryonic and larval lethal), wild type, sick (phenotypes other than the above two), and unknown. For genes annotated with more than one phenotype, the most severe one (assuming lethal>sick>wild) was chosen as their phenotypes.

The degrees of genes in the protein physical interaction network were retrieved from the Human Protein Reference Database (HPRD) [24]. To compare the function distribution of the genes in different categories, we used Gene Ontology (GO) Biological Process for protein function annotation. Gene Ontology annotations of 12,715 human genes were downloaded from NCBI [40] and the classifications based on biological processes were used. Similar to Zhou et al. [41], a GO node is referred as *informative *if it covers more than 500 genes, and none of its descendant nodes cover that many genes. 25 GO informative nodes were defined according to the criterion. To test whether UEHGs, disease genes or other genes were over/under represented in each of the 25 function categories, we used hyper-geometric distribution to calculate the p-value.

Gene length information was retrieved from UCSC genome table browser [42]. All the genes were first mapped to their refSeq IDs for length information retrieval. To assess the significance of the difference in the length of genes in different categories, Wilcoxon rank sum test was used to calculate the p-value.

In the process of collecting various features, some genes were not annotated in certain databases. We limited our comparisons to genes with information. The number of genes included for each comparison can be found in the corresponding tables or figures. For more information on the method and materials, see Additional file 1.

## Authors' contributions

ZT conceived of the study, performed the three-way classification, carried out the comparison of the evolutionary rate on amino acid level, protein physical interaction degrees, and drafted the manuscript. LW carried out comparison on all the other features. MX studied the relationship of gene expression correlation with protein essentiality. XZ, TC and FS jointly guided this research. All authors read and approved the final manuscript.

## Supplementary Material

Additional File 2 **List of UEHGsThe list of UEHGs **The list is contained in this file. Click here for file

Additional File 1 **Supplementary materials **Details of methods are described in this file. Click here for file

## Figures and Tables

**Table 1:** Comparison of evolutionary rate among three groups of genes

	UEHGs			Disease genes			Other genes		
	Mean(SEM)	Median	Range	Mean(SEM)	Median	Range	Mean(SEM)	Median	Range

Ka	0.053 (0.0015)	0.033	0–0.48	0.080 (0.0017)	0.061	0–0.70	0.092 (8.0E-4)	0.066	0–1.37
Ks	0.58 (0.0047)	0.57	0.076–2.0	0.63 (0.0043)	0.61	0.14–2.4	0.66 (0.0022)	0.62	0.041–4.2
Ka/Ks	0.088 (0.0023)	0.059	0–0.59	0.12 (0.0023)	0.10	0–0.70	0.13 (0.001)	0.11	0–1.2

Ka, the number of non-synonymous substitutions per non-synonymous sites. Ks, the number of synonymous substitutions per synonymous site. Ka and Ks values are calculated based on human-mouse orthologous pairs as described in the main text. The first column in each group is the mean followed by standard error of the mean (SEM).

**Table 2:** Cross-species comparison of gene essentiality between human and *S. cerevisiae*.

	**Total**	**Essential**	**Non-essential**	**Unknown**
**Homologs of UEHGs**	384 (20.2% of UEHGs)*	138 (35.9%) (7.3% of UEHGs)*	**242(63.0%)**	**4(1.1%)**
**Homologs of disease genes**	196 (9.8% of disease genes)*	51 (26.0%) (2.5% of disease genes)*	**142(72.5%)**	**3(1.5%)**
**Homologs of other genes **	1005 (3.5% of other genes)*	379 (37.7%) (1.3% of other genes)*	**618(61.5%)**	**8(0.8%)**
**No homologs**	**4641**	**505 (10.9%)**	**3427(73.8%)**	**709(15.3%)**
**Total yeast genes**	**6179**	**1058 (17.1%)**	**4397(71.2%)**	**724(11.7%)**

Yeast genes are mapped to three human gene groups by homologous mapping. The first three rows are for each human gene group and the fourth row is for yeast genes without human homologous genes. The last row summarizes all the yeast genes. The first column lists the number of homologous genes found for each human gene group and the fractions with respect to the number of human genes in the corresponding group (show in brackets marked with *). The following columns list the number of yeast essential, non-essential, and unknown genes for each human gene group. The fractions with respect to the number of homologs found in each group are shown in brackets. In the second column, the fraction of homologous essential genes over the human genes in each group is given in the bracket with *.

**Table 3:** Comparison of the length of various parts of UEHGs, disease genes, and all other genes.

	**UEHGs (n = 1400)**	**Disease (n = 1773)**	**Others (n = 10304)**	**P-value**
**Coding sequence length**	1501 ± 38 1109	2205 ± 73 1537	1849 ± 15 1459	3.2E-09
**Total exon length**	2545 ± 48 2136	3250 ± 78 2557	2752 ± 18 2343	1.6E-06
**Number of exons**	10.7 ± 0.2 8	13.5 ± 0.3 10	9.9 ± 0.1 7	9.6E-07
**Total intron length**	35698 ± 1558 15588	60376 ± 2836 23528	54881 ± 1139 18540	0.012
**5' UTR length**	546 ± 22 238	582 ± 21 251	560 ± 8 245	0.55
**3' UTR length**	559 ± 21 247	569 ± 21 243	575 ± 8 254	0.86

For each row the first line gives the average value ± s.e.m, and the second line gives the median. UTR stands for untranslated region. Pair-wise rank sum tests are performed and only the largest P-values are listed in the last column.
