# Supplementary material for: Further understanding human disease genes by comparing with housekeeping genes and other genes
Source: BMC Genomics. 2006 Feb 21;7:31. doi: 10.1186/1471-2164-7-31 (PMC1397819; doi:10.1186/1471-2164-7-31)
Supplement: Additional File 1 — Supplementary materials Details of methods are described in this file. [file 1471-2164-7-31-S1.doc]

### Supplementary

**Compile disease gene list**

Disease genes were obtained from OMIM (Online Mendelian Inheritance in Man, URL is http://www.ncbi.nlm.nih.gov/entrez/query.fcgi?db=OMIM). There were 3,962 records in the morbidmap (Jun 6, 2005) and we restricte to entries with known sequence (OMIM ID marked with *), with known sequence and phenotype (OMIM ID marked with #) and with phenotype description, molecular basis known (OMIM ID marked with +). After filtering, we obtain a list of 2,012 genes with unique OMIM IDs.

**Compile ubiquitously-expressed genes list**

Ubiquitously expressed genes were obtained from the gene expression experiment results by Su^1^. The data are publicly available for download at [http://symatlas.gnf.org](http://symatlas.gnf.org/). The overall expression level is 776.5 standard Affymetrix average difference units, and we choose genes with expression level greater than 550 standard units in at least 73/79 tissues, these genes are treated as ubiquitously-expressed human genes (briefly as UEHGs). A total of 1,789 such genes are collected and the list is available online.

We don’t use 200 units as described in Su et al. 2002 since the data are different. In 2002 experiment, Su et al. used human (U95A) chips and for the 2004, HG-U133A array and customer designed chips were used. The expression level generated by the above two platforms are different. The mean expression for 2002 data is 311.33 units and median is 75 units. For the 2004 dataset, the average expression is 716.05 and median is 235. We determine that 200 units will only be good for 2002 dataset.

For yeast, ~17% of the genome is essential, and for C.elegans, the fraction is about 10%. We estimate ~10% human genome is essential which equals roughly 2,000 genes. With this number in mind, we try to find appropriate criteria for defining our UEHGs. We decide to allow some missing expression in a small fraction of tissues being examined. Instead of requiring UEHGs being expressed in “ALL” the tissues, now we require expression in “almost all” the tissues. We chose 73 tissues (roughly 90%) as cutoff for that purpose. Given expression in at least 73 tissues and the set of UEHGs is about 2,000, the cutoff value for expression level can be determined to be ~550.

In order to show that our results don’t heavily rely on a specific criterion for defining UEHGs, we tested with another criterion. We then repeated our analysis on the evolutionary rates. The new criterion is that expression level at 300 and expressed in all 79 tissues, this generates a set of UEHGs of 2,038 genes. The overlap with old sets is 1,509 genes, quite large as expected.

The result is similar to what we observed previously, only the P-values slightly changed (Fig S1). Therefore it shows that our findings are not sensitive to specific criteria.

P-values are listed below

| Ks | Disease | Other |
| --- | --- | --- |
| UEHG | 3.09E-13 | 2.69E-25 |
| Disease |  | 5.50E-04 |

| Ka | Disease | Other |
| --- | --- | --- |
| UEHG | 1.75E-30 | 9.26E-54 |
| Disease |  | 9.09E-05 |

| Ka/Ks | Disease | Other |
| --- | --- | --- |
| UEHG | 3.46E-30 | 2.91E-46 |
| Disease |  | 2.26E-04 |

**Human other gene list**

We use NCBI Entrez human gene database as annotation of human genome^2^. There are nearly 33,000 human genes in Entrez. By excluding all the UEHGs and disease genes that we collected, a list of 29,129 genes is defined as “other genes”. The total number of other genes is not very informative in most cases. When features are collected for each gene, many genes don’t have the desired information. Comparisons are performed only on genes with information and the number of genes that are included for comparison is listed in the corresponding tables and figures. The only case that the number of other genes is used is in Table 2 and Fig 3, where the fraction of the homologous genes for other genes and the fraction of the essential homologous genes for other genes are calculated.

**Mouse orthologs and calculation of Ka, Ks and Ka/Ks**

The mouse homologs and corresponding Ka, Ks of totally 15,726 human genes are pre-calculated and downloaded from NCBI HomoloGene (<ftp://ftp.ncbi.nih.gov/pub/HomoloGene/build41.1/>). For human genes with more than one mouse homologs, we exclude them for further consideration due to paralog issues (175 such cases). Among the 15,726 human genes, 1,591 are successfully mapped to UEHGs list, 1,736 are mapped to disease gene list, and 12,171 are categorized as others. To test the statistical significance of the difference of Ka, Ks and Ka/Ks distributions among the three groups, a Kolmogorov-Smirnov test is used to calculate the p-value.

**Comparison of our gene sets and Huang’s gene set**

We compare our list of UEHGs, disease genes and other genes with the gene set used by Huang et al.^3^ so we can tell how much the two are overlapped. We obtained the original gene list from Dr. Kim Fechtel through personal communication. They considered 843 disease genes, 783 can be successfully mapped to our genes. 734/783 are mapped to our disease genes, 78 are mapped to our UEHGs, and 48 are mapped to our other genes. They also considered 10,655 other human genes. 9,290 of them can be successfully mapped to our genes. 7764/9290 are mapped to our “other genes” and 578 of them are mapped to our disease genes and 1,101 are mapped to UEHGs. Since their other genes contains many UEHGs, that might explain why their results are not in consistent with ours.

**Conservation of nucleotide and amino acid**

To study the conservation at the nucleotide/amino acid level for different genes, we use the results of a recent research based on 8 species multiple-sequence alignment^4^. Conservation scores are downloaded from UCSC Genome Browser website (http://hgdownload.cse.ucsc.edu/down

loads.html#human) on Aug 20, 2005. We only focus on the conservation of coding regions. To calculate the conservation score for an amino acid, we take the average of the conservation scores of the three nucleotides which correspond to the codon for the amino acid. We also retrieve the human sequence variation information from Swiss-Prot protein knowledgebase (<http://us.expasy.org/sprot/sp-docu.html>). The original amino acid positions are mapped to nucleotide positions on the corresponding chromosome to obtain the conservation score.

**Length of different parts of genes**

To compare the length of various parts of UEHGs, disease genes, and all other genes, we obtain the length data from UCSC genome table browser (<http://genome.ucsc.edu/>)^5^. Genes are first mapped to refSeq IDs, 1,783 out of 2,012 disease OMIM IDs can be mapped to refSeq IDs and we can retrieve 1,773 length information for them. 1,423 out of 1,789 Unigene IDs can be successfully mapped and 1,400 can obtain length information. We also compile a list of 23,833 refSeq IDs which don’t overlap with above two sets and 10,304 of them can retrieve the length information.

**Human protein-protein interaction**

The protein physical interaction degree information is obtained from Human Protein Reference Database (HPRD)^6^. XML files are parsed using java program to obtain the protein symbol and interaction information. Disease genes’ OMIM IDs are mapped to Human Gene Nomenclature (HGNC) official symbols and 1,723 can be linked in HPRD. 1,280 UEHGs can be mapped in HPRD and all the other proteins having official symbols (9,481) are put together as the third group. Due to the large fraction of proteins have no records of any interaction, we decide to use only those proteins have at least one interaction for the analysis.

***C.elegans* homolog and RNAi Phenotype**

After using similar method as described above to identify human homologs and map UEHGs and disease genes, we divide totally 20,448 C.elegans genes collected by NCBI into four groups: 695 UEG homologs, 480 human disease gene homologs, 2669 non-UEG/disease gene homologs and 16688 other genes that don’t have human homologs. By the WormMart tool of WormBase (<http://www.wormbase.org/>), RNAi phenotypes of *C.elegans* genes are retrieved using WormBase gene sequence name. We then map WormBase sequence name into NCBI gene based on Entrez Gene ID. We divide the RNAi phenotype into four categories: lethal (include both embryonic and larval lethal), wild type, sick (known phenotypes other than the above two), and unknown. For genes with more than one phenotype, we choose the most severe one, assuming lethal>sick>wild in their severity.

**Yeast homolog and phenotype**

We divide 6,179 yeast genes collected by NCBI into four groups: 384 UEHG homologs, 196 human disease gene homologs, 1,005 non-UEG/disease gene homologs and 4,641 other genes that don’t have human homologs. The yeast gene deletion phenotype data are downloaded from *Saccharomyces* Genome Database (SGD, <ftp://genome-ftp.stanford.edu/>). We consider three phenotypes: lethal, nonlethal and unknown.

**Human gene function annotation**

Gene Ontology annotations of 12,715 human genes are downloaded from NCBI (ftp://ftp.ncbi.nlm.nih.gov/gene/DATA/) on June 7^th^ and we consider only the biological part of the annotation. For these genes, 1331 can be mapped to UEG, 1741 can be mapped to disease genes and 9,814 are put together as others. Similar to the definition used in Zhou et al.^7^, a GO node is referred as *informative* if it covers more than 500 genes, and none of its child nodes covers the such many genes. We divide all the biological processes into 25 function categories according to the informative nodes. To test whether UEHGs, disease genes or other genes are over/under represented in each of the 25 function categories, we use hyper-geometric distribution to calculate the p-value. In the figure, colors are used to present the logarithm of the p-value.

**Weighted least square linear regression**

Since the variances among different groups are not constant (as shown in Fig 6), we applied weighted linear regression. In weighted linear regression, we try to minimize. $\omega_{i}$is the weight for the i-th group and it’s defined as , $n_{i}$is the number of genes in i-th group, $y_{i,j}$is the observed Ka/Ks for j-th gene in i-th group. $\hat{y}_{i,j}$is the estimated Ka/Ks based on the regression model $\hat{y}_{i,j}\sim\alpha+\beta x_{i}$. $x_{i}\in(2,...,6)$ is the numerical representation of onset ages group. For more information on weighted least square linear regression, readers can refer engineering statistic handbook section 4.4.5^8^.

**References**

1. Su, A.I. *et al*. A gene atlas of the mouse and human protein-encoding transcriptomes. *Proc. Natl. Acad. Sci. USA* **101,** 6062-6067 (2004).
2. Maglott, D., Ostell, J., Pruitt, K.D. and Tatusova, T. Entrez Gene: gene-centered information at NCBI. Nucl. Acids Res. **33**, D54-D58 (2004).
3. Huang H, Winter E.E., Wang H., Weinstock K.G., Xing H., Goodstadt L., Stenson P.D., Cooper D.N., Smith D., Alba M.M., et al. Evolutionary conservation and selection of human disease gene orthologs in the rat and mouse genomes. *Genome Biol*, **5**, R47 (2004).
4. Siepel, A., Bejerano, G., Pedersen, J.S., Hinrichs, A.S., Hou, M., Rosenbloom, K., Clawson, H., Spieth, J., Hillier, L.W. Richards, S. et al. Evolutionarily conserved elements in vertebrate, insect, worm, and yeast genomes. *Genome Res*., **15**, 1034-1050 (2005).
5. Karolchik, D. *et al*. The UCSC Table Browser data retrieval tool. Nucl. Acids Res. **32**(Suppl 1), D493-D496 (2004).
6. Peri, S. *et al.* Development of human protein reference database as an initial platform for approaching systems biology in humans. *Genome Res*. **13**, 2363-2371 (2003).
7. Zhou X., Kao M.J. & Wong W.H. Transitive functional annotation by shortest-path analysis of gene expression data. *Proc. Natl. Acad. Sci. USA* **99**, 12783-12788 (2002).
8. Engineering statistic handbook, URL [http://www.itl.nist.gov/div898/handbook/index.htm].

**Supplementary figure and table**


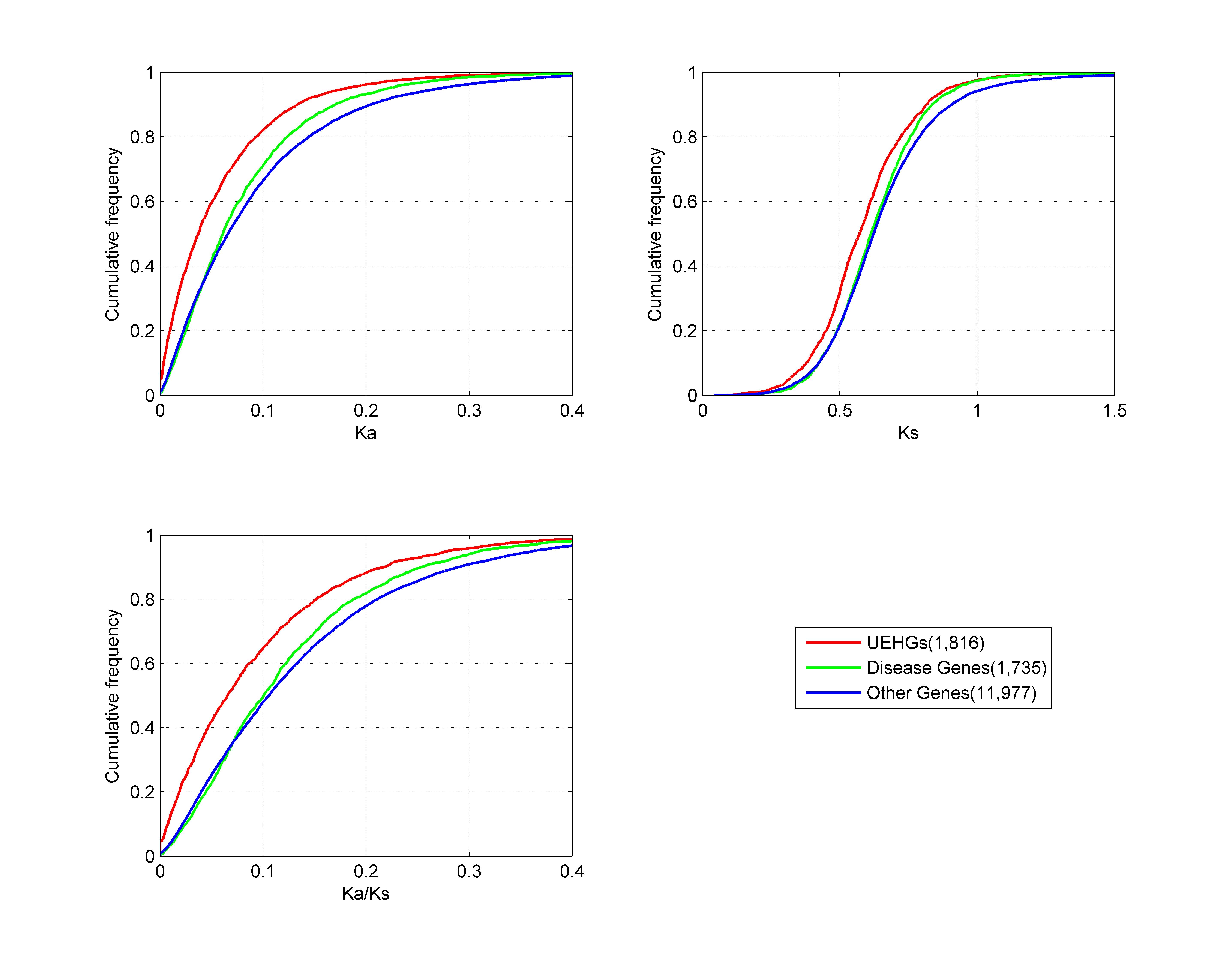


Fig S1. The cumulative distribution functions of Ka, Ks and Ka/Ks of UEHGs, disease genes, and other human genes. UEHGs are defined by the new criteria as described above. Ka and Ks are calculated based on human-mouse orthologous pairs. Ka, the number of non-synonymous substitutions per non-synonymous sites. Ks, the number of synonymous substitutions per synonymous site.


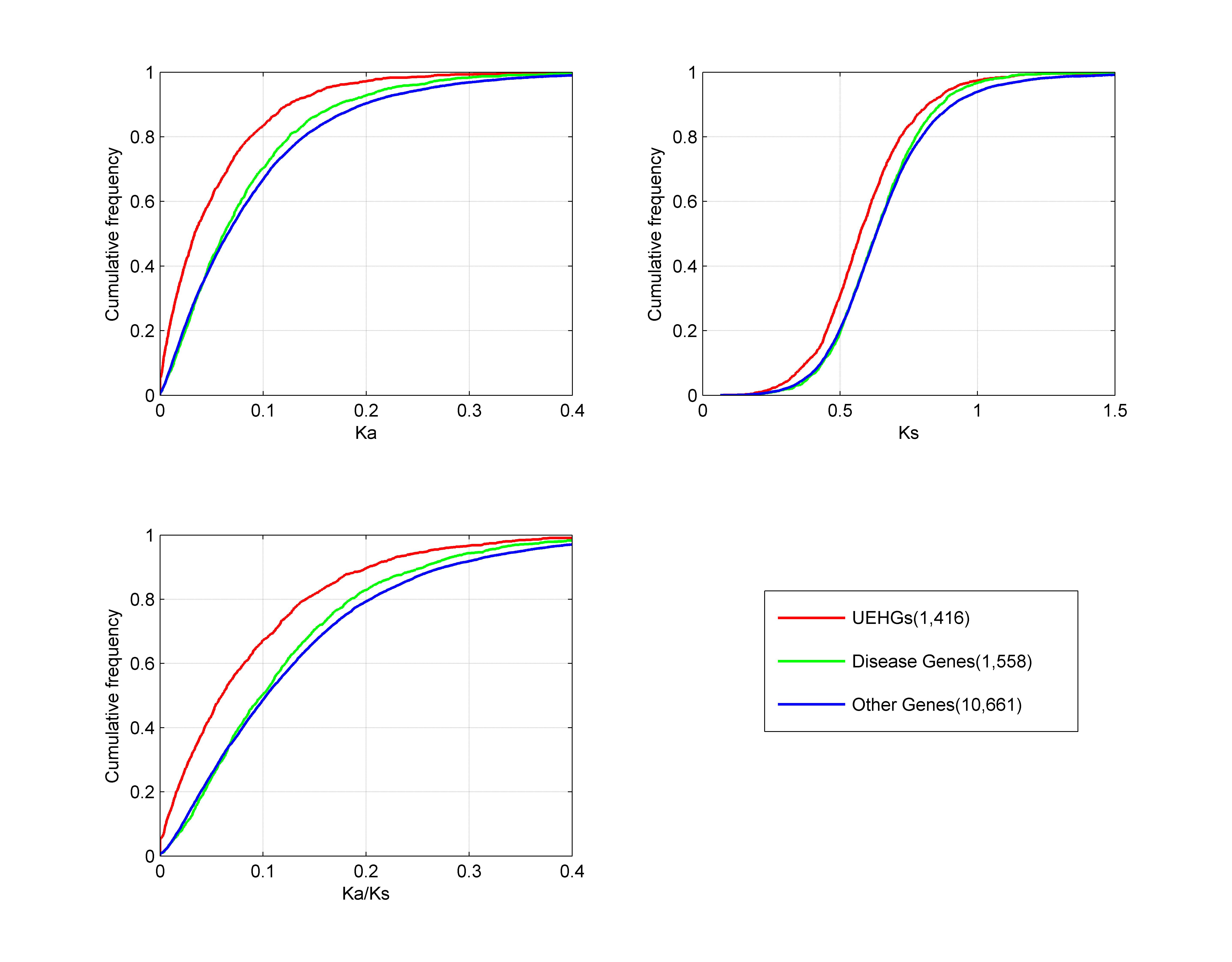


Fig S2. Cumulative distribution function curve of Ka, Ks and Ka/Ks for UEHGs, disease and all other genes. The Ka and Ks are calculated based on the human-rat orthologous pairs. Ka, the number of non-synonymous substitutions per non-synonymous sites. Ks, the number of synonymous substitutions per synonymous site. Different groups of genes are represented in different color as list in the legend, the number of genes in each group is also listed.
